# Nanostructured Organic/Hybrid Materials and Components in Miniaturized Optical and Chemical Sensors

**DOI:** 10.3390/nano10030480

**Published:** 2020-03-07

**Authors:** Mario Prosa, Margherita Bolognesi, Lucia Fornasari, Gerardo Grasso, Laura Lopez-Sanchez, Franco Marabelli, Stefano Toffanin

**Affiliations:** 1 Institute of Nanostructured Materials (ISMN), National Research Council (CNR), via P. Gobetti 101, 40129 Bologna, Italy; mario.prosa@cnr.it (M.P.); margherita.bolognesi@cnr.it (M.B.); 2 Plasmore s.r.l., viale Vittorio Emanuele II 4, 27100 Pavia, Italy; lfornasari.plasmore@gmail.com (L.F.); llopez.plasmore@gmail.com (L.L.-S.); 3 Institute of Nanostructured Materials (ISMN), National Research Council (CNR) c/o Department of Chemistry, ‘Sapienza’ University of Rome, Piazzale Aldo Moro 5, 00185 Rome, Italy; gerardo.grasso@ismn.cnr.it; 4 Physics Department, University of Pavia, via A. Bassi 6, 27100 Pavia, Italy; franco.marabelli@unipv.it

**Keywords:** integration, smart-system, portable, sensors, biodiagnostics, optical, optoelectronics, electrochemical, analytics, organics, nanoplasmonics

## Abstract

In the last decade, biochemical sensors have brought a disruptive breakthrough in analytical chemistry and microbiology due the advent of technologically advanced systems conceived to respond to specific applications. From the design of a multitude of different detection modalities, several classes of sensor have been developed over the years. However, to date they have been hardly used in point-of-care or in-field applications, where cost and portability are of primary concern. In the present review we report on the use of nanostructured organic and hybrid compounds in optoelectronic, electrochemical and plasmonic components as constituting elements of miniaturized and easy-to-integrate biochemical sensors. We show how the targeted design, synthesis and nanostructuring of organic and hybrid materials have enabled enormous progress not only in terms of modulation and optimization of the sensor capabilities and performance when used as active materials, but also in the architecture of the detection schemes when used as structural/packing components. With a particular focus on optoelectronic, chemical and plasmonic components for sensing, we highlight that the new concept of having highly-integrated architectures through a system-engineering approach may enable the full expression of the potential of the sensing systems in real-setting applications in terms of fast-response, high sensitivity and multiplexity at low-cost and ease of portability.

## 1. Introduction

Sensors have become increasingly important in our daily lives. Home pregnancy tests, blood glucose meters, gas-leak sensors are some among the multitude of possible examples. However, the continuous growth of the global population in conjunction with the need for better standards of living are pushing for a rapid development of new technologies of sensing. The scientific interest is even more clear when considering the ca. 20,000 research articles on this topic that have been published last year (bibliographic sources: Web of Science and Scopus by searching for the word “sensor” in the title of articles and review papers).

In this context, biochemical sensors represent a fascinating class of sensing systems that has provided a change of paradigm in analytical chemistry and microbiology, passing from general analytical systems to dedicated systems [1]. The chemical information at the basis of the sensing effect is obtained in real time, possibly on site, as a result of the interaction between sensor and chemical and biological agents in a two-step process: recognition and signal processing. Because of a chemical or physical reaction/interaction with a sample, a change in a physical property occurs or is observed (remote sensing) in the sensor receptor. In cases where the chemical recognition in the receptor does not directly modify an electrical property (resistance, potential), but rather other properties like heat, mass, or light changes, some type of transduction is required to obtain an electrical signal compatible with the electronic circuits.

Depending on the signal-detection technique, sensors can be divided into different types such as resistive, catalytic, thermoconductive, electrochemical and optical [2,3]. Every class has its strengths and weaknesses. However, a lot of parameters must be considered in evaluating sensor performance: sensitivity, selectivity, stability, response time, accuracy, durability, maintenance, portability, cost, safety and lifetime [4]. As a remarkable example, we mention that electrochemical methods allow a signal that is proportional to the analyte concentration to be obtained, thus guaranteeing easy-to-calibrate sensors in a simple configuration. However, lowering the limit of detection is the major goal in the realization of electrochemical sensors, while rapid response-time, low-cost, ease of operation and potential for miniaturization have already been achieved [5]. 

Among others, selectivity—the ability to respond primarily to the analytes in the presence of other species—is another key issue with sensors, which can be achieved physically, by the selective interaction of the analyte with electrostatic or electromagnetic fields, or chemically, using an equilibrium-based or kinetically-based selective interaction with the layer containing the (bio)reagents [6]. By contrast with electrochemical sensors in which analyte specificity is an open issue [7], optical sensors allow for a highly selective analysis in real-time and automated operational configuration. Moreover, the suitable combination of label-free and multiplexing detection with the reduction of the susceptibility to environmental interference is the next expected breakthrough in the engineering of optical sensors. 

Even though they are endowed with different characteristics, biosensors based on electrochemical and optical methods can aim at becoming powerful and robust analytical tools: nanostructuring the sensing active region for amplifying the collected signal and/or implementing functional nanomaterials for increasing analyte specificity are considered valuable strategies [3,5].

Finally, the concept of sensors can be exploited even more effectively if included in miniaturized instrumentation that makes it possible to convert the functions of an entire lab into user-friendly analytical instruments [8]. Highly integrated systems such as lab-on-a-chip (LOC) devices have shown themselves to be highly effective for laboratory-based research, where their superior analytical performance has established them as efficient tools for complex tasks in genetic sequencing, proteomics, and drug discovery applications [9]. Although the chips themselves are cheap and small, they must be generally used in conjunction with bulky detectors (especially in the case of optical sensors), which are needed to identify or quantify the analytes or reagents present. This feature prevents the use of these systems in point-of-care or in-field applications. Furthermore, most existing detectors are limited to analysis of a single analyte at a predetermined location on the chip.

The lack of an integrated and versatile detection scheme (one which is miniaturized, selective and able to monitor multiple locations on the chip) is a major obstacle to the deployment of diagnostic devices in the field and has prevented the development of more complex tests where rapid, kinetic or multipoint analysis is required.

In this scenario, we remark that an increasing trend among academics and in research and development (R&D) is towards monolithic-integrated sensor systems, which merge optical, electrochemical, optoelectronic and electronic elements (e.g., light sources and/or detectors) into one functional unity fabricated on one common substrate to build LOC systems. In particular, the great interest of the scientific community on organic/hybrid light-emitting and light-sensing devices that has characterized the last few decades has led to technologies mature enough to be exploited in real-setting applications. 

Innovation in system engineering is, however, useless if not supported by advancements also in the synthesis and use of functional materials. The evolution of the technology requires indeed a combinatorial development of material science and engineering. Despite a multitude of review articles that have thoroughly described specific classes of organic and hybrid (multicomponent) materials and composites for sensing applications [10,11,12,13], here we focus on reporting about the advancements, the figures of merit and the effectiveness of different multipurpose integrated detection schemes in biosensors with the objective of inspiring readers towards a new way of visualizing devices as constituting elements of multimodal and miniaturized sensing platforms. 

In particular, we report on the use of organic and hybrid nanostructured materials in optoelectronic, electrochemical and plasmonic elements of biosensors for contaminant detection and/or biodiagnostics. The cross-correlation between structural characteristics and functional properties of these classes of nanomaterials plays a major role in the definition and realization of next-generation portable and wearable sensors, if the architectures of the single-component devices are suitably engineered. 

For this purpose, the review is organized as follows: in Section 2 we report notable examples of effective miniaturized and conformable light sources and detectors based on organic and hybrid devices; Section 3 describes the nanostructuring of organic and hybrid materials in 2D and 3D geometries for achieving smart-detection schemes in both optical and electrochemical biosensors; finally, Section 4 reports on the assembling of the constituting single-component devices into portable and flexible sensors for wearable contaminant detection and biodiagnostics.

## 2. Light-Sources and -Detectors Based on Organic/Hybrid Nanostructured Materials and Architectures

Organic and hybrid materials are widely applied in optoelectronic and photonic components and devices because of the fascinating possibility to tune their physical-chemical properties for an improved performance with respect to the corresponding bulk inorganic counterparts. In particular, thanks to the targeted design, synthesis and processing of new nanostructured materials and architectures, great progress in the realization of optoelectronic and photonic components and devices has been recently reported, such as the new generation of miniaturized, easy-to-integrate, highly performing photodetectors, light-emitting devices, light-sensors and imagers, optical fibers, plasmonic and photonic structures [14]. This progress has triggered great advances also in the engineering and development of new detection schemes as, for instance, in the fascinating class of optical sensors. 

Optical sensors rely on the modification of an optical stimulus, in intensity, frequency and/or polarization, upon interaction with a target analyte. Optical modification (transduction) can occur through different mechanisms such as bioluminescence, photo- and electroluminescence, and surface plasmon resonance (SPR). In all these methods, light-emitting and light-sensing devices are fundamental parts of the sensor. In this section, a brief illustration on the use of nanostructured organic and hybrid materials for realizing light sources and light detectors is given.

**Figure 1:**
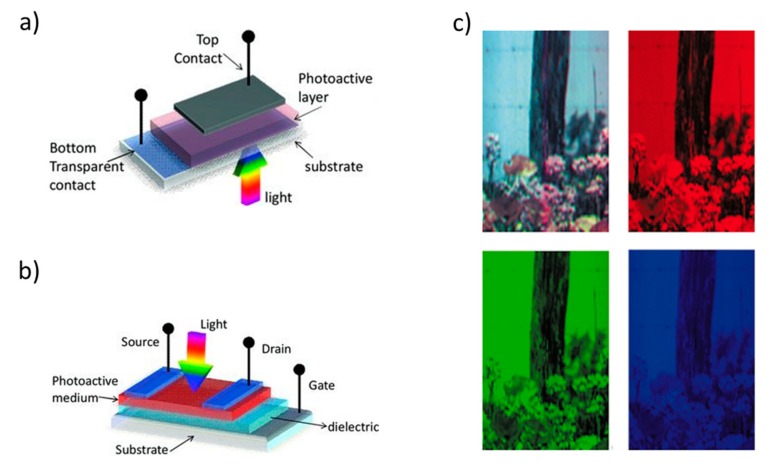
Typical device configurations of photodetectors: (**a**) a photodiode with vertical geometry illuminated from the bottom-side and (**b**) a phototransistor with the light window entrance on the top. Reproduced with permission from [30] © 2020 by WILEY-VCH Verlag GmbH & Co. KGaA, Weinheim. (**c**) full-color image (with the corresponding monocolor, R, G and B images) taken by an organic photodetector (OPD) array. Adapted with permission from [31] © 2020 by WILEY-VCH Verlag GmbH & Co. KGaA, Weinheim.

Within photodetectors, the geometrical layout of the active layers and electrodes define some performance characteristics of the sensors. Photodetectors such as photoconductors and phototransistors are based on a lateral structure with (at least) two electrodes in a side-by-side geometry (Figure 1). These photodetectors, due to the micrometric electrode spacing, typically show a slow response at relatively high driving bias (but with switching and amplifying functionality, in the case of transistors). Photodetectors based on a vertical structure, where the active layer is sandwiched between vertically stacked electrodes, include photodiodes (PDs) and photomultipliers (PMs) (Figure 1). These photodetectors have smaller electrode vertical spacing with a short carrier transit length, which generally provides a fast response speed at relatively low driving bias, with small noise.

Within ultraviolet–visible–near-infrared (UV–vis–NIR) photodetectors, the simplest are PDs typically based on inorganic semiconductors such as silicon (Si), gallium arsenide phosphide (GaAsP), gallium phosphide (GaP) and InGaAs (indium gallium arsenide). Despite the good electrical properties, standard inorganic light sensing systems have a number of limitations that restrict their application. For example, silicon has poor mechanical flexibility [15]. In addition, high carrier mobility and long lifetimes can lead to crosstalk between neighboring pixels in image sensors [16]. Also, with broadband absorption, filter-less color discrimination is hard to be achieved [17]. These limitations can be partially overcome with organic and hybrid photodetectors.

Organic and hybrid photodetectors has seen outstanding progress thanks to the advance in the research and development on new nanostructured organic and hybrid materials for light-sensing and light-converting devices, such as third-generation photovoltaic (PV) devices which can be comprised by hybrid dye-sensitized solar cells (DSSC) [18], organic photovoltaic devices (OPVs) [19,20,21], quantum dot PVs (QD PVs) [22], perovskite photovoltaic devices (PeroPV) [23,24]. PDs based on new nanostructured semiconductors such as organic semiconductors, metal-halide perovskites and quantum dots, combine simple processing (i.e., solution-based), flexibility and conformability, programmable optoelectronic properties through chemical engineering, easy integration onto complementary metal oxide semiconductors (CMOS), and high performance [25]. Thanks to these properties, new PDs designs and architectures can be envisaged for applications outside traditional technologies, such as biomedical devices i.e., large-area and flexible UV–vis–NIR light and X-ray imagers [26], artificial retinas [27,28], machine vision and robotics, endoscope-based imaging [29] (Figure 1). In particular, organic semiconductors are ideally suited for interfacing with biological systems. Indeed, their mechanical and chemical nature offers better compatibility with tissues and suits the non-planar form factors often required for biomedical implantable sensors.

With reference to specific optical and electrical characteristics, organic semiconductors present an effective combination of higher extinction coefficient and lower dielectric constant [32] which must be considered when comparing them with respect to the inorganic counterparts. Thus, the amount of material used to absorb light, in comparison with silicon, can be reduced down to sub-micrometric thin films (in a compromise with sensitivity), enhancing conformability, flexibility and the capability to be miniaturized. On the other hand, to overcome the high exciton binding energy (due to the low dielectric constant) and the low charge-transport properties of organic semiconductors (1 cm^2^ V^−1^ s^−1^), organic and hybrid heterojunctions of two (electron donor and acceptor) different semiconductors are used into the same active region, thus allowing a high photocurrent, that is correlated to high dynamic range in luminous signal detection. This enables the tailoring of the absorption range of the optical sensors from the UV to the deep infrared. 

Typically, highly conjugated molecular or polymeric semiconductors are used in heterojunction organic photodetectors (OPDs), thanks to their high conductivity and band-gap in the visible region. Within the different strategies for the molecular design of conjugated polymers and small molecules for optoelectronic applications, the push-pull approach is one of the most widely applied in literature. This strategy consists in combining, through covalent binding, electron-rich and electron-deficient moieties into the same structure: electron-rich units such as thiophene, bithiophene, fluorene, carbazole, dibenzosilole, benzodithiophene and their derivatives can be bound with electron-deficient units such as quinoxaline, benzothiadiazole, diketopyrrolopyrrole, isoindigo, etc. and their derivatives. Conjugation between the repeating units can be modulated through the design of side functional atoms and groups and through the nature of the covalent spacer between units, allowing the fine tuning of electronic properties (such as charge mobility and bandgap). This fine molecular design allows to obtain a plethora of medium-to-low band gap semiconductors [21]. 

Organometallic complexes are also effectively used as electron donor p-type semiconductors in heterojunction OPDs [33]. Indeed, metal ions can strongly alter the electronic and optical properties of organic conjugated molecules. For example, the presence of transition metal centers can promote the formation of triplet excited states, significantly enhancing exciton lifetime and diffusion, allowing in turn high OPD photocurrent. Indeed, metal (i.e., copper (II), Al (III), Zn (II)) phthalocyanine complexes exhibit excellent charge transport characteristics. Copper phthalocyanines are particularly well suited for red light, high-speed OPDs, while other metal-phtalocyanine-based OPDs are more suited for NIR OPDs, due to their typical absorption in the 600–700 nm range. 

In turn, C_60_ or C_70_ fullerenes and their derivatives, perylene-diimmide and bisimmide polymers and derivatives can be used as electron accepting materials [34,35,36]. In particular, the strong absorption bands of C_70_ and perylene derivatives in the visible range can improve the spectral range and external quantum efficiency (EQE) of OPDs.

Thanks to the wide variety of organic and hybrid materials available, different choices can be done in relation with the target application, sensing method or analyte to be detected (for more examples please refer to Table 2 of Ref. [37]).

Regarding color selectivity, the benchmark for OPDs is a quasi-Gaussian spectral response with a full-width- at-half-maximum (FWHM) ≤ 100 nm. This can be achieved by appropriate design and coupling (with minimized spectral overlap) of semiconducting organic materials, and/or by the manipulation of the electro-optics of the device [38]. The use of thick active layers and of selective contacts (electron-/hole-blocking layers) in OPD can lead to dark currents as low as 10^−9^ A cm^−2^ and to a signal-to-noise ratio (S/N) competitive to that of silicon-based PDs. Also, the specific detectivity of OPDs, which can be defined as the S/N of a PD with an effective area of 1 cm ^2^ irradiated with an optical power of 1 W and detected at a bandwidth of 1 Hz, has reached and overcome that of silicon and GaP (10^15^–10^14^ J) [32,39,40]. The highest operational light intensity range of an OPD, described by the linear dynamic range (LDR), is 9 orders of magnitude (180 dB) for visible, and 12 orders of magnitude for UV [41,42,43], which is comparable with that of GaP, GaAsP and Si photodiodes (220 dB) [41]. Finally, OPDs have been also demonstrated to reach temporal bandwidths as high as 400 MHz in the visible range, making these devices useful building blocks for organic photonic integrated circuits for imaging and sensing.

As for light detection, the research on innovative semiconductors such as organic materials, metal-halide perovskites and quantum dots and on the design of new device architectures has been the driving force for the progress of light-emitting devices for sensing applications.

Considering optical sensors for reliable in-field applications, lasers, inorganic light-emitting diodes (LEDs) and broad-spectrum lamps are the well-assessed light-sources used in literature. Lasers have the advantage of a high-power, collimated, narrow bandwidth and possibly frequency pulsed light emission. This allows efficient and selective excitation of targets to be achieved [44,45,46], but their application is unadaptable and restricted due to high costs, need for eye protection, bulky size and a limited selection of wavelengths. Inorganic LEDs and micro-LEDs as light sources are highly efficient, and arrays of LEDs can achieve large-area excitation on targets allowing even fluorescence imaging applications [47], although the emission uniformity of micro-LEDs arrays is limited, due to the point-like source nature of LEDs [48]. 

Broad-spectrum discharge lamps can be color-tuned with color filters (for selective excitation of absorbing or photosensitized targets), but undesirable side-effects due to heating which can interfere with measurements remain critical [49]. Therefore, for all applications where it is needed a light source with a sufficiently high irradiance (in the order of mW/cm^2^), and a spatially controlled emission at a selected wavelength, new solutions have been proposed.

Organic light emitting diode (OLED)-based light sources are robust, portable, miniaturized, cost-effective and fast [50,51]. In addition, thanks to their soft-mechanical and chemical nature, and their low dimension, OLEDs can be easily interfaced with biological and environmental systems, while minimizing their perturbation, and they can be easily integrated into industrial value chains allowing for a real in-line and on-line process control. 

To date, the OLEDs technology is already at an industrial stage thanks to the huge progress in OLED-based displays. The use of organometallic complexes based on noble metals such as platinum, iridium and osmium has guaranteed a wide color tunability (covering the whole visible and NIR range) and a high stability in operating conditions [52,53]. Red and green OLEDs easily reach lifetimes up to 10^6^ h, while only recently blue OLEDs have shown improved device lifetimes up to 10^3^ h [54]. OLEDs can reach incredibly high performance parameters such as a high-brightness at low driving voltages (10^6^ cd m^–2^ at ≈ 8 V and 10^4^ cd m^–2^ at ≈ 3 V) [55]. Blue OLEDs still cannot reach such high performance parameters, but they have been recently demonstrated to show a brightness >10^3^ cd m^−2^ at the same low voltages and EQE as red and green OLEDs [56]. In addition, the use of phosphorescent organometallic complexes and thermally activated delayed fluorescent (TADF) emitters has enabled to obtain OLEDs showing an internal efficiency (IQE, electron-to-photon conversion) of nearly 100% [57,58]. Most recently, also the substitution of noble metals-based organometallic complexes as emitters with more abundant and convenient metals (i.e., copper or zinc) is object of many studies and will allow the increase in the volume of production and the reduction of costs both of raw materials and of processes [59,60]. 

**Figure 2:**
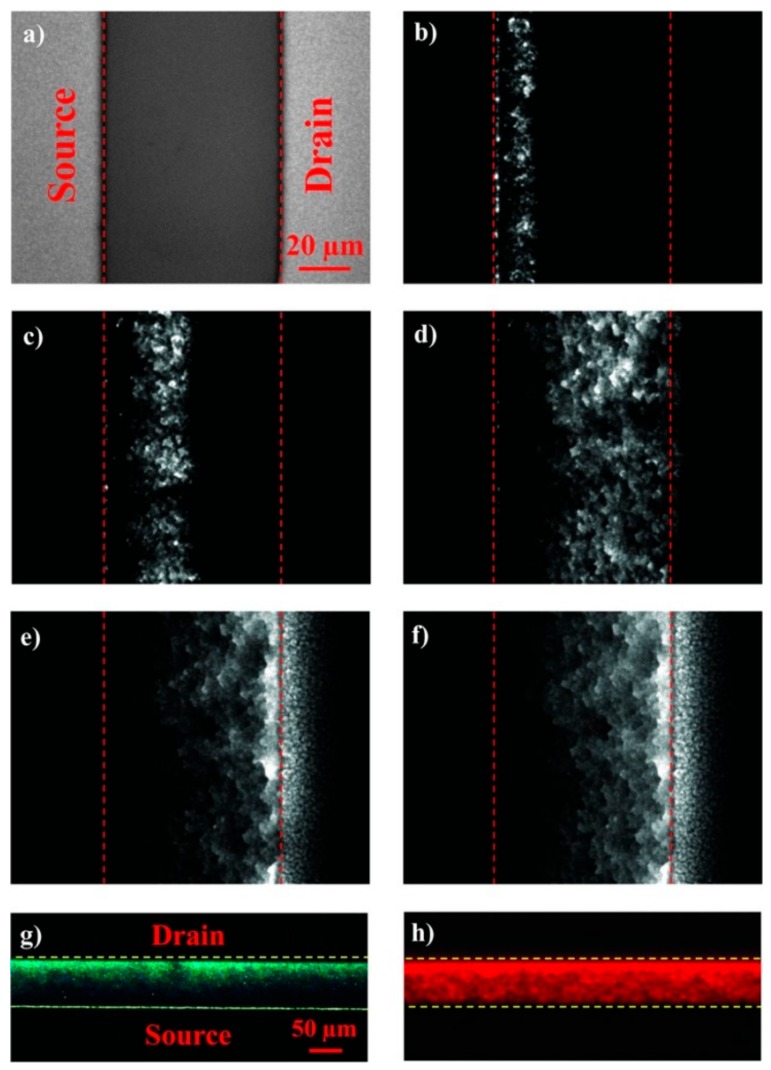
Images of the light-emitting area in the channel of an organic light-emitting transistor (OLET). A transmission optical microphotograph of the unbiased device is reported as the reference (**a**). The gate-source bias (V_GS_) is varied at increasingly high voltages: −20 V (**b**), −40 V (**c**), −60 (**d**), −80 V (**e**) and −100 V (**f**), while the drain-source bias (V_DS_) is kept constant at −100 V. Illuminated channels of green- (**g**) and red- (**h**) emitting OLETs are reported, with recombination layers based on different organic active materials. Reproduced with permission from [65] © 2020 by WILEY-VCH Verlag GmbH & Co. KGaA, Weinheim.

If the class of light sources is broadened by considering multifunctional devices, organic light-emitting transistors (OLETs) are emerging as optoelectronic component capable of integrating the electrical properties of a transistor (electrical amplification, switching…) with the light-generating capacity and color tunability [61]. A clear advantage of OLETs, inherent in the structure of the device, is the higher quantum electroluminescence efficiency, compared to the corresponding OLEDs [62]. This feature is correlated to the lateral charge transport and in-plane radiative charge recombination which are intrinsic to the device architecture [63]. The as-realized planar µm-wide emission region in the channel of the transistor enables the integration of the light-emitting component with photonic planar structure [64], thus opening the way to the realization of highly integrated optical communications and optoelectronic systems. In the application field of miniaturized and integrated optical sensors, the use of intense nanoscale light sources with controlled light-emission pattern and tunable photonic integration in optoelectronic systems is a key enabling step for the development of effective low-cost, compact, portable and highly sensitive sensors (see Section 4). In this regard, in OLET devices not only can the light-emission zone be shifted across the transistor channel but also the lateral dimension can be tuned by acting on the gate voltage of (Figure 2) [65]. This peculiar feature with respect to OLEDs holds promise to be exploited for defining innovative excitation and detection schemes in next-generation optical sensors by fine-tuning the optical pig-tailing within the photonic components in a planar architecture [14].

OLEDs and OLETs often share a similar composition of the active layer, which follows the specific requirements of their sensor application (for more details, see also Section 4 of the present review). As a first example, label-free biosensors such as photoplethysmogram (PPG) sensors, extracting the oxygen saturation level from the difference in light absorbed by oxy-hemoglobin and deoxy-hemoglobin, work with at least two coupled light sources emitting in different spectral regions. To this aim, polyfluorene co-polymers can be effectively used as cheap and efficient building blocks for obtaining emissive layers with colour-tunable emission, depending on the co-monomer used (i.e., benzo-*2,1,3*-thiadiazole for green, hexylthiophene-benzothiadiazole for red [66]). As a further example, muscle contraction sensors, which rely on a different sensing mechanism, need for light-emitting sources operating in the red-NIR regions, for allowing the light to penetrate into tissue for centimeters and return to detectors. This has been achieved, for example, by using the efficient conjugated polymer Superyellow (SY) [67]. Finally, for time-resolved luminescence sensors based on frequency modulated OLEDs, the use of a spike- and tail-free emitter in the UV-blue spectral region, such as a carbazol-biphenyl derivative instead of the more classical molecular host-guest emissive layer such as coumarin-doped Alq_3_, has resulted in more reliable sensors [68].

OLEDs and OLETs have been also demonstrated to reach a high-speed response, at a modulation frequency even higher than that of commercially available LEDs. In detail, a peak brightness of ≈10^6^ cd m^−2^ at a 40 MHz modulation frequency under 10 ns pulse operation has been recently obtained in OLETs [69]. Thus, these devices are perfectly suited for the realization of photonic integrated circuits for imaging and sensing. Finally, high-frequency modulation has allowed also a large improvement in the electroluminescence properties of the most recent light-emitting transistors based on solution-processed hybrid perovskite emitters (PeLEFET) [70]. Alternated-current operation (AC) of PeLEFET at frequencies in the 10^5^ Hz range has enabled an increase in their brightness by two orders of magnitude, enabling their operation at room temperature.

## 3. Nanostructured Components and Materials in Miniaturized Detection Schemes

In this section we will provide an insight into the detection methods that are enabled by the use of nanostructured materials and might then be integrated into the architecture of the sensor to guarantee high degree of miniaturization together with suitable sensitivity and selectivity for in-field and real-time applications. Given the very broad topic of this contribution, we focus our attention on the detection schemes based on optical probing (and specifically on the high-performing methods related to surface-plasmon resonance effects), but also on non-optical methods such as biochemical and electrochemical methods whose component constituents, however, belong to the same category of nanostructured organic and hybrid materials which has been presented in the previous section. In this way, the reader will have a more general and exhaustive view of the range of possible applications for this category of multifunctional materials.

### 3.1. Analyte Detection Based on Plasmonic Systems

Nanomaterials show a modified interaction pattern which has significant effects on the macroscopic features of the system, giving rise also to non-conventional behaviors [71]. Nanostructured materials are currently applied in various fields, including healthcare (in targeted drug delivery, regenerative medicine, and diagnostics) [72], energy harvesting [73,74], photovoltaics [75,76], cosmetics [77], gas sensing [78,79,80], electronics [81], environmental protection and food supplements [82]. From the point of view of sensing application, this evolution triggered an impressive development in the performance and miniaturization of sensing devices which are nowadays pervasively affecting our everyday life.

In many respects, this huge progress has been fostered by the evolution of optoelectronics and photonics, i.e., the possibility of manipulating light–matter interaction and light behaviour. Nowadays, the extremely large availability of miniaturized and well controlled light sources and detectors opened the way to very compact, ease of use, cheap and robust optical devices. Then, as observed in communication technologies, where an almost complete transition from electric-based to photon-based transmission occurred, optical sensing gained more and more importance. With respect to electrical signals, photons are less affected by electric- or magnetic- fields and self-interference effects. In general, the main detection mechanism can be described as follows: once a definite optical resonance has been identified (this can be related to an excited state of a molecule or a material or to a cavity mode), one is measuring the change induced in such a resonance by the presence of the target analyte. The change can be the direct switching on (or off) of the optical excitation; this is the case for fluorescence emission of some dyes in the presence, or otherwise, of the target molecule. In the more general case, the change occurs in the wavelength, the phase, the polarization or the angular dispersion of the optical signal. In this case, the effect is a combination of features of both device material(s) and nano-structuring. Nano structuring is also responsible of an enhancement effect of the material optical response via the localization of the photonic electromagnetic field. It is such an enhancement controlled by nanostructuring which is exploited to increase the sensitivity and allow the measurement of small analyte concentrations. Apart from SERS (surface-enhanced Raman spectroscopy), which is a real tool to identify molecules (but requires a spectral scan), the described scheme of detection is not specific for any given analyte without a recognition tool.

This can be the “labelling” of the target molecule e.g., by its binding with a known dye. Actually, the most part of photonic based sensors belongs to the so called “label-free” category, i.e., the detection is not depending on a previous selection of the analyte in the sample examined.

On the one hand, this is an advantage because the same detection scheme can be applied to detect a large panel of molecules. It is the bare presence of the molecule, with its mass and contribution to refractive index which is detected. On the other hand, a functionalization step is required to make the device able to select the specific target molecule. This is the very tricky point because one has to find a specific bio-chemical recognition mechanism for each analyte and provide its activation within the active region of the sensor.

Considering optical components and devices, nanostructured systems can be divided into two categories: those based on metallic behavior, concerning plasmonic excitations, and those built with dielectric components, paving the way to the realization of photonic devices.

The first class of devices exploit the properties of metals, mainly gold and silver, but also metallic alloys and heavily doped semiconductors or oxides [83]. The choice is driven by low losses criterium, but easy of fabrication and stability is playing a big role (e.g., silver shows better performance, but, due to oxidation affecting it, gold is often preferred). As for the photonic systems, a large variety of dielectric materials, from semiconductors to insulators, is considered. The most used is silicon and silicon-based compounds like silicon nitride. This is due to the compatibility with the microelectronics industry and the well assessed fabrication technologies. Nevertheless, besides silicon dioxide, also titania oxide structures are studied, which offer a good compatibility with e.g., biomolecules [84].

In this respect, interest in organic, polymeric materials and the possible related structures is relevant too. Besides the usual features characterizing polymers (low cost of material and fabrication, flexibility, lightness, …), organic materials add in general a high compatibility with biological matter [85,86,87].

The features of the plasmonic and photonic systems are quite complementary and a new interest emerged in combining them into hybrid systems to take advantage of the best properties of both. Generally speaking, photonic systems have a much better-defined spectral response that can be finely tailored and is intrinsically stable and robust. On the other side, plasmonic systems offer a superior performance in terms of field confinement and enhancement, combined with the drawback of relatively large losses and a much broader spectral response [88]. Up to now, even considering that sensitivity is also depending on the chosen optical parameter (shift of spectrum, polarization, phase, intensity, in reflectance, transmittance and fluorescence mode), field enhancement remains the main factor affecting the final optical detection performance. Then, metallic based plasmonic systems offer the most suitable platform [89]. This is true even for fluorescence, provided that a thin dielectric layer is inserted between the metal and the dye to avoid fluorescence quenching [90,91]. Actually, fluorescent-based methods are, in general, the most sensitive tools used for detection of small quantities of substances in different matrices. On the other hand, they are heavily subjected to matrix effects and also need the use of fluorescent markers, which are provided by complex pretreatments of the sample to be analyzed. These often require laboratory-grade analytical techniques and are not suitable for integration “on chip”. In other words, these are typical “label” techniques.

Among label-free methods, a relevant position is occupied by SPR.

SPR sensors exploit the excitation of charge waves: (i) in metallic nanoparticles (localized surface plasmon resonance (LSPR) [92], (ii) at the surface of metal layer (surface plasmon polariton (SPP)) or (iii) a combination of both. The detection mechanism is based on the change in the spectral- and/or angular- distribution of the optically excited mode, when the refractive index of the medium close to the surface is changing. Despite the fact that LSPRs can be easily optically excited, they exhibit an intrinsic spectral broadening which makes them less sensitive to variation in the medium refractive index.

**Figure 3:**
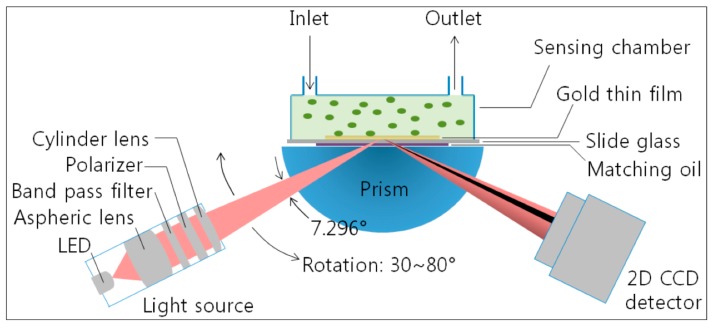
Scheme of a surface plasmon resonance (SPR) system in Kretschmann configuration. © 2020 MDPI, reproduced with permission from ref. [94].

By contrast, SPP are longitudinal waves characterized by a shorter wavelength than the electromagnetic wave having the same frequency. Consequently, some coupling strategies must be used to optically excite SPP. The largest part of SPP based SPR systems typically measure the attenuated total reflection (ATR) in the Kretschmann configuration, where a thin (less than 50 nm) layer of gold is deposited on a prism [93]. This implies a careful control of either angle of incidence and beam collimation, and then the use of costly and bulky optical setup and mechanics, not easy to miniaturize (Figure 3).

In order to make the system simpler and, to some extent, more portable, an excitation through an optical fiber design has been developed. Drawbacks in this case are the need of stable laser sources and the cost of the disposable fiber sensors. A more extensive and complete review of these classes of instruments is given in ref. [95].

A different coupling strategy is offered by a periodic nanostructuring of the plasmonic surface. When the period of the nanostructure is comparable to the wavelength of the optical mode, diffraction effects (either of light and polaritons) allows the direct excitation of SPP at an arbitrary angle of incidence and even at normal incidence. This greatly simplifies the optical system even though it is at the cost of losing angular resolution, which is the most sensitive parameter used in commercial instruments for the detection of molecular interaction on the surface.

A further advantage of using nanostructured surfaces is the easy implementation of imaging capabilities. Image analysis implies that each portion of the active surface can be considered as an almost independent sensor, opening the way to multiplexing, i.e., the simultaneous detection of a whole panel of different analytes [96].

Some works have been published about systems based on grating of holes in a gold film [97]. The majority of them report on spectral/intensity light-modulation in a transmittance configuration in order to benefit of the extraordinary transmittance properties of such periodic surfaces (i.e., gratings). However, a great disadvantage of such configuration originates from the light path across the fluidic cell which is necessary for providing the sensitive surface with the analytes to detect: evidently, this detection scheme affects the measurement by reducing the sensitivity and introducing interference effects.

To disentangle the optical signal collection from the fluidic system, one should collect the light reflected by the grating from the (transparent) substrate side: however, this approach requires an effective cross-talking between plasmonic modes at the exposed (metal/fluid) surface and the back (metal/substrate) interface. Actually, this can be easily achieved considering the local excitations supported by nanostructuring. As a matter of fact, a hole in a metal behaves like a mirror-like metallic nanoparticle in a dielectric and exhibits a series of localized plasmonic excitations. In principle, these LSPRs are dispersionless: however, when they are active in the same spectral region where SPP occurs, hybridized modes between the two kind of excitations take place.

**Figure 4:**
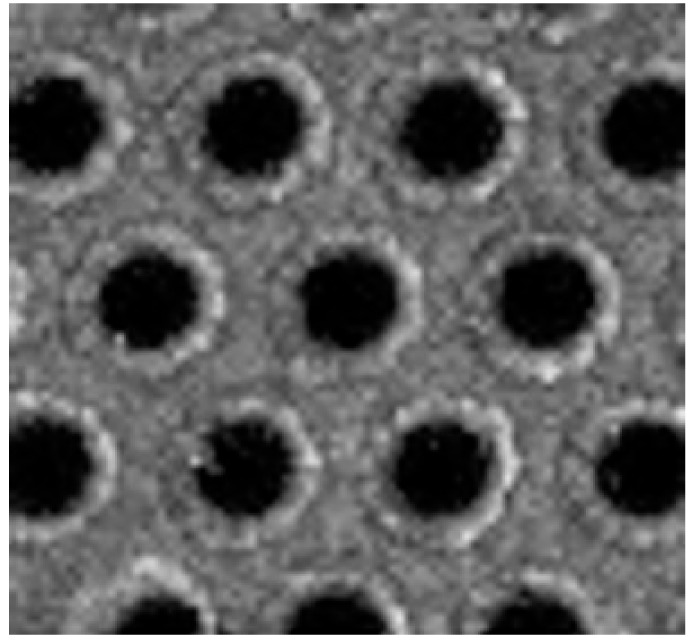
Scanning electron microscope (SEM) image of a typical nanostructured gold layer produced by Plasmore S.r.l., with a hexagonal plasmonic lattice (lattice pitch = 500 nm). Top of polymeric pillars corresponds to the black areas.

In order to better illustrate the interplay of all these effects, we can use as an example a specific nanostructured metal-dielectric grating, produced by Plasmore S.r.l, which might be implemented for a sensitive detection in a reflectance configuration. The system is constituted by a hexagonal lattice of polymeric pillars embedded in a relatively thick gold layer (about 150 nm). A scanning electron microscope (SEM) view of a typical surface so obtained is shown in Figure 4. The gratings are prepared through colloidal lithography and plasma etching techniques. The detailed fabrication protocol was presented by Giudicatti et al. [98]. In this context it is worth noting that the colloidal mask fixes the lattice pitch, while the etching procedure determines the pillar dimensions and shape, which also give a significant contribution to the optical response [99].

**Figure 5:**
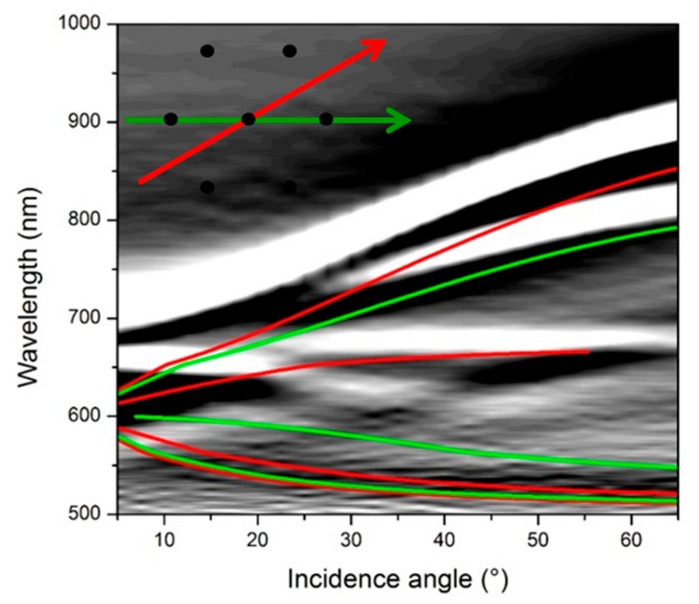
Second derivative of the spectral reflectance plotted as a function of the incident angle. The bright and dark signals correspond to minima and maxima in the reflectance spectrum, respectively. The superimposed red and green lines correspond to the calculated plasmonic dispersion for two orientations of the hexagonal lattice.

A good tool to explore the dispersion behavior of plasmonic excitation is to study the response as a function of the incident angle. Figure 5 shows the complicated interplay occurring among dispersionless localized modes and propagating polaritons in surfaces with a lattice pitch of 400 nm. Indeed, in the figure the theoretical dispersion of polaritons for the two main symmetry directions of a hexagonal lattice at the gold/glass interface is also shown (red and green lines), while the opening of a propagation gap at the folding point corresponding to zero incidence-angle is clearly visible. The crucial point is the spectral and modal superposition of polaritonic and localized modes by using suitably designed planar grating. The folding process of the polaritonic modes is controlled by the pitch of the grating, whereas the spectral position of the localized modes is mainly dictated by the size and the shape of the nanostructured elements forming the grating, namely, the diameter of the holes in the metal layer.

**Figure 6:**
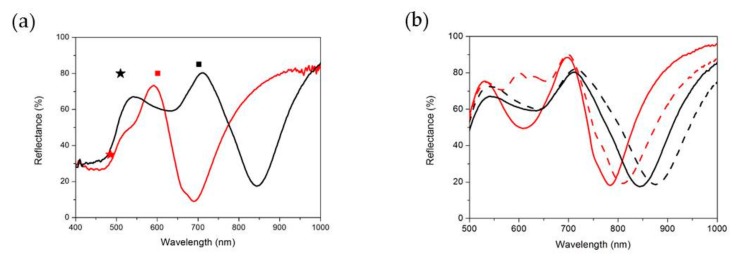
(**a**) Reflectance spectra for two gratings having lattice pitch of 500 nm (black line) and 400 nm (red line). The stars and the squares point out the calculated gold/air and gold/glass polariton wavelength, respectively. (**b**) Solid black and red lines correspond to the reflectance spectra recorded for two gratings having the same lattice pitch (500 nm) but different hole size. Dashed lines show the spectra acquired for the two samples when water is dispensed on the grating surface. These graphs are obtained from unpublished data from the authors.

Figure 6a shows the reflectance measured from the back through the glass substrate of two gratings with different pitches. The hole dimension has been scaled to the pitch in order to have a similar relationship with the polariton frequency. The scaling effect of the whole response is evident, with the reflectance maximum corresponding to the folding gap shifting from 700 nm to 600 nm when passing from the 500 nm to 400 nm lattice pitches.

As a general constraint upon the integration of the plasmonic-sensing surfaces into a working optical sensor, it is mandatory to tune the resonance wavelength of the grating to the emission specifications of the used light-source. In view of a fully effective system miniaturization of the complete sensor, the use of conformable easy-to-adapt planar or stripe-like light-emitting sources (as in the case of organic and hybrid light-emitting diodes and transistors, see Section 2) may play key-role in the engineering of portable and flexible plasmonics-based sensors.

The competitive advantage of these nanostructured plasmonic devices with respect to other standard detection schemes is the possibility to design and control the resonance localized modes by acting on the geometrical features of the gratings. Indeed, the hole size can be tuned by increasing or decreasing the etching time. Hole size also plays a role in defining the spectral response. As shown in Figure 6b (solid lines), by modulating the cavity diameter between 340 and 300 nm, it is possible to shift the reflectance peak wavelength of the grating from 850 nm to 770 nm.

It is worth noting that, despite reflectance spectra having been taken from the backside, through the substrate, they carry information about the top surface. Hence, by changing the medium on the top side from air to water, resonance minima in reflectance shift towards higher wavelengths (dashed spectra in the figure). It follows that spectra are sensitive to refractive index changes in the medium above the surface.

Indeed, this is the detection principle of this kind of detectors: any adhesion of molecules at the exposed surface changes the local refractive index close to surface and hence is inducing a spectral shift of the whole plasmonic resonance (the broad deep in reflectance spectra).

In the case of small refractive-index changes, and then small spectral shift, the change can be also detected as an intensity change in the reflectance at a given wavelength close to the resonance minimum. Thus, it is possible to build up sensors based on quasi-monochromatic light sources and a camera as light detectors [100]. By working in imaging modality, it is possible to be sensitive to the spatial positioning of the intensity change. In biosensing applications this finding allows the end user to monitor multiple interactions simultaneously.

The crucial point is related to the functionalization process. The functionalization of plasmonic nanostructures is much more challenging than that of continuous metal films. Nanostructured gratings often comprise multiple materials (e.g., glass and gold) and exhibit surface curvatures (e.g., edges, tips). On the other hand, the heterogeneous chemistry composition of nanostructures could be exploited to functionalize only the areas where the electromagnetic field is enhanced rather than the entire surface [101].

The simplest and straightforward functionalization method is the passive adsorption of receptors to the metal surface. It is also possible to covalently bind an array of different biological molecules through simple functionalization strategies as self-assembled monolayers (SAMs) which use thiol-containing compounds attached to the gold component of the plasmonic grating. However, these approaches often result in a uncontrollable release of receptors and high nonspecific adsorption of complex matrixes to the surface. The ideal functionalization method is expected to create a functional coating that: provides optimized concentration of receptors, preserves their biological activity and good accessibility and guarantees a low non-specific binding of non-target molecules.

In the development of diagnostic applications for the screening of complex matrixes of interest, as serum or milk, it is appropriate to follow a functionalization strategy that comprises the use of highly hydrophilic polymers (carboxymethyl dextran, polyacrylamide derivatives…). In this way, the functionalization layer of the grating guarantees high stability during large number of regeneration cycles and low fouling properties [102]. As an example, we mention the commercially available copolymer MCP-2F (Lucidant Polymers) for the coating of a wide range of materials (glass, silicon oxide, silicon nitride, gold, PDMS, COC, and Teflon).

The MCP-2F is a poly(dimethylacrylamide) copolymer that incorporates a silane moiety and it is functionalized with nacryloyloxysuccinimide (NAS). The NAS moiety extends the succinimidyl ester from the surface. This group is highly reactive toward nucleophiles such as amines naturally present in most of the target molecules.

A film of the copolymer MCP-2F bearing active esters was successfully grafted by a combination of physical adsorption and covalent linking to the plasmonic nanostructure of Plasmore. It is worthwhile noticing that Plasmore plasmonic gratings have been used for example for the detection of long pentraxin 3 (PTX3), a biomarker for different human pathologies and infections [100] and for the detection of mycotoxins in barley and beers [103].

### 3.2. Analyte Detection Based on Electrochemical Bio/Chemosensoristic Devices

Selectivity and sensitivity are key parameters for the assessment of analytical performances of sensing devices, especially to evaluate their application for real matrix analyses and for multianalyte detection purposes. Optical sensors generally show a good sensitivity and selectivity against the analyte(s). However, a proper design of optical sensors based on the use of nanostructured materials and the adoption of suitable sample pretreatments (e.g., dilution and analyte extraction) can substantially improve the performance and sensitivity of various optical sensors. Similarly, the introduction of nanotechnologies into research and development in electrochemistry is nowadays addressing important issues in sensoristic field. Nanophase materials possess peculiar physical, electronic and chemical properties (compared to bulk materials) that can be exploited for the functionalization of electrode surfaces both as ‘direct’ active layers (e.g., in non-enzymatic electrochemical sensors) and interfacing layers between an electrode surface and biological recognition element (e.g., enzymes and antibodies). The use of nanomaterials to modify electrode surfaces can strongly affect the analytical performance of non-enzymatic electrochemical sensors through changes of operating parameters including working potential, surface morphology, signal amplification and catalytic efficiency.

In electrochemical sensors, both selectivity and sensitivity are strongly affected by the material of working electrode and modification of surface architecture. The chemical modification of the electrode can be achieved with nanostructured materials e.g., to increase electrode surface area, to enhanced electron transfer kinetics or to enhance selectivity and or sensitivity to the analyte(s) [3,104].

In this section, the introduction of the classes of nanomaterials used in the optimization of the performance of electrochemical devices is meant to broaden the overview of the status of progress of the engineering of sensors for real-setting applications. Furthermore, the cited nanomaterials could be also used in new and interesting applications based on the combination and the integration of electrochemical and optical techniques, as in spectroelectrochemistry approaches [105,106], in the fabrication of optically transparent electrodes and in bio/chemosensing applications [107] with enhanced selectivity, sensitivity and signal intensity. In this context, a recent analytical integrated approach for innovative monitoring and diagnostics of the environment and the agro-food supply chain is provided by the patented physicochemical sensing device called ‘Snoop’ [108]. The physico-chemical device ‘Snoop’ uses one or more tailor-made designable advanced chemical or biological ‘sensitive materials’ (SMs) (included nanomaterials) in the same sensor. The interaction with one or more target analytes (or substances belonging to the same chemical class) with selected SMs can induce specific or aspecific physicochemical (electric or optical) responses.

**Figure 7:**
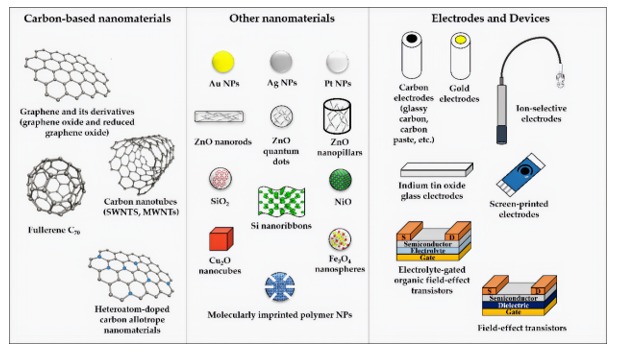
Schematic illustration of carbon allotrope nanomaterials and other nanomaterials together with electrochemical and electronic tools described in this review.

Referring to classes of innovative multifunctional nanomaterials, carbon allotrope nanomaterials (Figure 7) are widely used in the fabrication of electrochemical sensors because of interesting features like an increased electroactive surface area (large surface-to-volume ratio and specific surface area), a faster electron transfer kinetics and an enhanced interfacial adsorption properties (exploited for adsorption of molecules and to reduce surface fouling effects) [109].

Graphene is the basic building block for other graphitic materials, which consists in a 2D single-layer sheet of sp^2^-hybridized carbon atoms structured into a honeycomb-like hexagonal pattern. Beside high surface area, graphene exhibits remarkable electrochemical properties (e.g., such as high electric conductivity, a zero-bandgap semimetal behavior, a large potential window), low charge-transfer resistance, excellent electrochemical activity and fast electron transfer rate. Three-dimensional graphene (3D graphene) has been recently used as support and stabilizer of bimetallic electrocatalyst NiCo_2_O_4_ for non-enzymatic detection of urea in urine samples [110]. The sensor has shown an excellent analytical performance in both neutral and alkaline pH conditions, rapid response (approximately 1.0 s) and stability (no significant loss in activity after four months of storage at room temperature). Reduced graphene oxide (rGO) is a suitable alternative to the pristine graphene and graphene oxide for interesting features: a chemical reduction by removal of oxygen functional groups increases its conductivity (compared to graphene oxide). In addition, chemical reduction produces several chemically active lattice defects sites (compared to pristine graphene), making rGO a promising candidate for active layers in electrochemical (bio)sensors [111].

Carbon nanotubes (CNTs) (single-wall carbon-nanotubes or SWCNTs, double-wall carbon-nanotubes or DWCNTs, and multi-wall carbon-nanotubes or MWCNTs) are a graphene-derived class of carbon allotrope nanomaterials that display metallic or semi-conducting electron transport (depending on the sheet direction about which the graphite sheet is rolled) and they are extremely attractive carbon allotrope nanomaterials for a wide range of electrochemical sensing [109]. A potentiometric sensor for *Escherichia coli* O157:H7 detection in milk samples and apple juice samples was described in literature. In this work, SWCNTs were used as ion-to-electron transducers in potentiometric measures [112]. In the last 30 years, the integration of carbon nanotubes and graphene into field-effect transistor (FET)-type nanobiosensors has increasingly gained interest in sensing as a promising more sensitive, and portable label-free, analytical solution. Through the modification and functionalization of the gate electrode and the semiconducting channel of field-effect transistor, new possibilities have been opened for the development of new SWCNT and graphene FET-based biosensors [113]. A recent example is the functionalization of gold gate electrode in an organic electrochemical transistor with poly(diallyldimethyl-ammonium chloride) + MWCNTs and graphene nanocomposites for the determination of gallic acid in green and black tea samples. The electrocatalytic activity of the gate electrodes was enhanced by MWCNTs and graphene, with the best detection limit (as low as 10 nM) in the case of MWCNTs nanocomposite [114]. In recent years, CNTs have been also gradually exploited for the manufacturing of new electroactive nanomaterials for solid-contact ion-selective electrodes (SC-ISEs) for the potentiometric determination of ions K^+^, Ca^2+^, H_3_O^+^, Pb^2+^, NH_4_^+^, NO_3_^−^ and ClO_4_^−^ also in real samples [115,116,117,118]. The application of nanomaterials in stack architectures is very promising for the development of high stable SC-ISEs with long operating lifetimes. [118] Several screen-printed electrodes (SPEs) modified with nanocomposites were also developed for electrochemical determinations of e.g., heavy metal and antibiotics in real environmental and food samples [119,120,121,122]. The use of SPEs modified by carbon black was also described for phenyl carbamate pesticides (carbofuran, isoprocarb, carbaryl and fenobucarb) detection in grain samples. The analytical performance of the sensor allowed a class-selective detection of several phenyl carbamates in food samples. The detection of carbaryl was possible for concentrations up to maximum residue limit levels (MRLs) [123]. Fullerenes and derived nanostructures [124] (e.g., nanorods) are very interesting nanomaterials in the electrochemical sensoristic field. A fullerene C_70_/polyaniline (a conductive polymer) nanocomposite modified glassy carbon electrode was recently exploited for the detection of pyridine herbicide fungicide triclopyr in spiked tomatoes extracted by square-wave voltammetry [125]. Heteroatom-doped carbon allotrope nanomaterials (e.g., doping with phosphorus and nitrogen) is another interesting family of nanomaterials that possess improved physicochemical and structural properties that can be used in electrochemical sensor devices [126,127,128,129].

Metal and metal oxide NPs have been largely used for the modification of solid electrodes, especially in association with carbon allotrope nanomaterials. Indeed, NPs can be dispersed in inorganic−organic nanocomposites, by conferring new remarkable synergistic electronic properties that cannot be achieved by individual nanocomponents [130].

Noble metal nanoparticles (AuNPs, AgNPs and PtNPs) are extensively employed to form nanocomposite with unique electronic and catalytic properties to be exploited in electrochemical sensors (Figure 7). The presence of AuNPs in rGO-AuNPs modified glassy carbon electrode had showed a positive effect in the preconcentration step in stripping voltammetry analysis for the detection of As^3+^ in soil samples [131]. Other recent examples of noble metal NPs-modified electrodes used in electrochemical sensors with the limit of detection in the nanomolar range include the differential pulse voltammetric detection of the insecticide methyl parathion in spiked water samples [132], the differential pulse voltammetric detection of Ca^2+^ in pork meat [133], the detection of antibiotic neomycin in spiked milk and honey samples [134] and for the determination of H_2_O_2_ in human serum and saliva samples [135]. The determination of synthetic azo-colorant Sudan I in real food samples (chili powder, chili, tomato and strawberry sauce) have been obtained by a voltammetric sensor based on a Pt/CNTs nanocomposite modified ionic liquid carbon paste electrode. The sensor showed a very large current response (thanks to an enhanced conductivity), a good selectivity, a biocompatible interface and a low limit of detection (LOD, 3 × 10^−9^ mol L^−1^) [136].

Zinc oxide (ZnO) is an inorganic semiconductor that forms several hierarchical nanostructures that have been recently exploited for electrochemical sensoristic purposes (Figure 2). ZnO nanopillars were recently used for coating gold electrode in direct chronoamperometric detection of Cd^2+^ in spiked river water samples (LOD = 3.6 × 10^−8^ mol L^−1^) [137]. An amperometric glucose biosensor with glucose oxidase physically immobilized onto ZnO nanorods showed a large linear range (0.05 mM to 1 mM) and good sensitivity (48.75 µA/mM) [138]. A gold electrode modified with ZnO quantum dots (QDs) has been recently used for voltammetric detection of mercury in groundwater samples (LOD = 2.5 × 10^−8^ mol L^−1^) [139]. Surface coating of electrode by ZnO helped electron migration between mercury and electrode surface and no sample pretreatments are required. Most QDs are inorganic semiconductor nanocrystal like ZnO nanocrystals or cadmium sulfide (CdS), whose electronic properties can be fine-tuned by varying the size of the nanostructures that can be used alone or as nanocomposite with CNTs e.g., for enzymes immobilization with higher direct electron transfer between the active site and electrode [140]. ZnO NPs co-doped with nickel and iron have been recently used for the fabrication of a FET sensor for the detection of hexahydropyridine in mineral water and tap water samples [141]. ZnO-CNTs- nanocomposites are peculiar nanomaterials that can strongly enhance the electroanalytical performance of modified electrode surfaces, especially in terms of charge transfer [142]. Some recent examples reported in literature include a carbon paste electrode modified by ZnO/CNTs nanocomposite for voltammetric determination of ascorbic acid in fresh vegetable juice, fruit juices and food supplement samples [138] and a screen-printed electrode modified with a gold NPs/graphene oxide nanocomposite for voltammetric detection of semi-synthetic β-lactam antibiotic cloxacillin in raw milk samples [143]. In the latter work, a pre-concentration step by addition of a cloxacillin-imprinted polymer to spiked milk samples was also described. Other recent electroanalytical application of pristine or metal oxide NPs nanocomposite include: NiO NPs-modified carbon paste electrode with the N-hexyl-3-methylimidazolium hexafluorophosphate ionic liquid for square-wave voltammetry measurement of p-nitrophenol in tap water, drinking water and river water samples [144], a glassy carbon electrode modified by Cu_2_O nanocubes/Ag NPs nanocomposite for impedimetric measures of hydrogen peroxide (H_2_O_2_) in spiked commercial milk [145]. The combination with Ag NPs improves sensitivity and linear detection range of the electrochemical measure, compared to analytical performances of other previously reported in literature H_2_O_2_ sensors based on pristine Cu_2_O. A carbon paste electrode modified with Fe_3_O_4_ nanospheres combined with a cobalt(II)-Schiff base complex was employed for square-wave voltammetric detection of NO_2_^−^ ion in spiked spring water, mineral water and tap water samples [146]. The newly synthesized cobalt(II)-Schiff base complex was involved in preconcentration of NO_2_^−^ ions at the electrode surface and no sample pretreatments were required.

Non-metal nanostructures and nonmetal oxide nanostructures provide effective surfaces and peculiar electronic characteristics that can enhance sensing performances. An electrode modified with nanosilica (nano-SiO_2_) has been recently described for the detection of Pb^2+^ in black tea and in wastewater samples. The nano-SiO_2_ characteristics of hydrophobic filler with a high specific surface area and a peculiar three-dimensional structure increase the number of Pb^2+^ binding sites. This can positively affect diffusion rates of target analyte(s), with possible involvement in the extraction step of ions at the electrode surface [147]. This modified electrode showed a good analytical performance (LOD = 7.3 × 10^−8^ mol L^−1^) and a long lifetime (2 months). Silicon nanoribbons have been exploited to make the semiconducting channel of a pH FET device for H_3_O^+^ detection in milk samples (from condensed milk powder) and bovine blood plasma. The developed proton exchange lipid bilayer membrane proved to be a resistant and highly efficient proton-conductor antifouling coating suitable for good analytical performances of the pH FET device in milk and bovine blood plasma samples (pH sensitivity of 64% and 35% per unit pH, respectively) [148].

Molecularly imprinted polymers (MIPs) are chemical artificial polymeric receptors with a tailor-made designable selectivity and specificity for a target analyte [149,150]. Impedimetric detection of estradiol in spiked commercial milk with no pre-treated sample has been described using Au NPs and molecular imprinted polymer electrodeposited on the surface of a glassy carbon electrode [151]. Magnetic NiO NPs decorated by molecularly imprinted polymer were used to modify the surface of a glassy carbon electrode for chronoamperometric detection of phenylurea herbicide chlortoluron in irrigation water samples [152].

An improvement in the molecular imprinting polymerization method is the synthesis of molecularly imprinted polymeric NPs. Thanks to higher surface-to-volume ratio and a better accessibility of recognition sites for the analyte, improvements in binding kinetics and detection sensitivity in MIP NPs-based sensoristic devices can be achieved [153]. An example of uses of MIP NPs modified carbon paste electrodes for electroanalytical detection of target analytes in real samples is the modification of carbon paste electrode composition for square-wave voltammetric determination organophosphate insecticide diazinon in well water and apple fruit samples [154].

## 4. Towards Smart Integration of Nanostructured Components for the Realization of Miniaturized Optical Sensors

In this section, we will focus on the system engineering of optical bio- and chemosensors through the implementation the organic and hybrid device components that we previously introduced. Particular attention is devoted to specific structural characteristics of the sensors such as miniaturization, portability and wearability that have a huge impact on the effectiveness of the use of the sensors in real settings. We aim at highlighting that optical bio- and chemosensors based on innovative organic and hybrid materials are expected to be well-suited to point-of-care and in-field applications.

**Figure 8:**
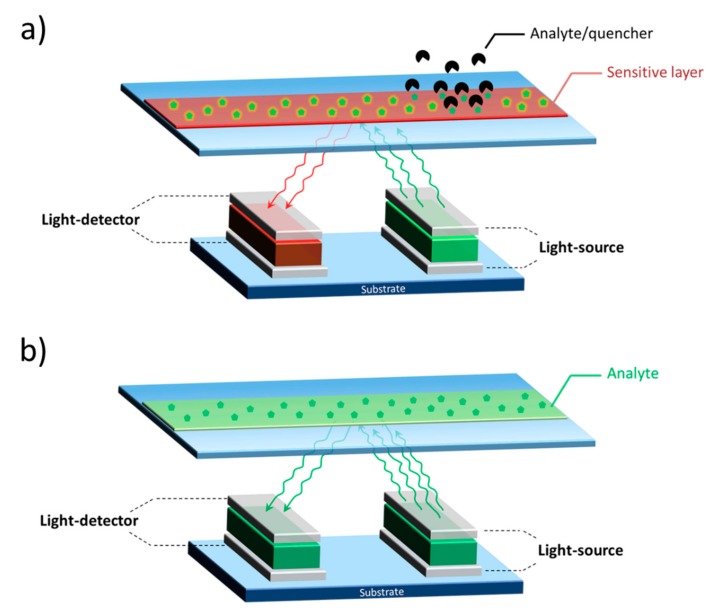
Schematic representation of optical sensors with a reflection-type architecture. The sensors comprise a light-emitting device, a light-sensitive device and a sensitive element, which acts as photoluminescent emitter sensitive to external quenchers (**a**) or absorber of the emitted light (**b**).

Integrated optical sensors are typically based on a light-emitting source and a light-detector, in conjunction with an element sensitive to the analyte of interest. In the working principle of the sensor, the light emitted by the source interacts with the sensitive element, where a signal variation occurs as a result of a bio/chemical event (Figure 8). The role of the photodetector is to monitor a spectral or intensity change of the light with respect to the original characteristics of the light emitted by the source. It is evident that, the three components must be endowed by compatible spectral characteristics.

Optical sensors are typically classified on the basis of the optical transduction modality (that is the working principle of the sensing element): (i) photoluminescence and (ii) absorption/transmission-reflection of light. In the first case the light interacts with the sensing element and hence is energetically modified (Figure 8a) while, in the latter case, the optical transduction involves a variation of the light intensity (Figure 8b).

One of the most common photoluminescence-based detection schemes relies on the dynamic luminescent quenching of the sensitive element, as a consequence of the presence of the analyte. That is, the emission of a dye is reduced in intensity by increasing the amount of the analyte that works as a quencher.

**Figure 9:**
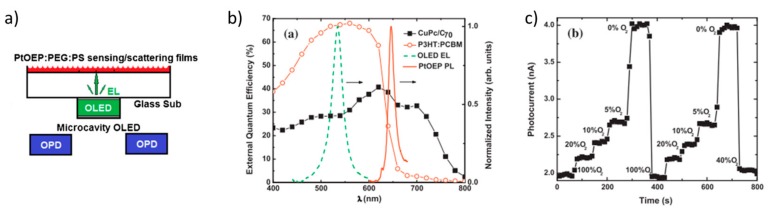
O_2_ sensor based on a back-to-back integration of a microcavity organic light emitting diode (OLED) and two organic light photodiodes (OPDs) by using Platinum(II) 2,3,7,8,12,13,17,18-octaethyl-21H,23H-porphyrin (PtOEP) in a polymeric matrix of PEG-PS as sensing element (**a**). The external quantum efficiency (EQE) of CuPc/C_70_ (black squares) and P3HT:PCBM-based OPDs (red dots) are reported with the EL (dashed green line) and PL (red line) characteristics of the OLED and the PtOEP sensing element, respectively (**b**). The signal of the O_2_ sensor is reported at different analyte concentrations (**c**). Reprinted from [156], © 2020, with permission from Elsevier.

By using that detection scheme, Shinar et al. attempted the realization of an oxygen-sensor by integrating an OLED as light-source, an a-(Si, Ge):H p-i-n diode as photodiode and a sensor film based on a polystyrene matrix doped with a metalorganic complex (Platinum(II) 2,3,7,8,12,13,17,18-octaethyl-21H,23H-porphyrin, PtOEP) [155]. Oxygen indeed represents an ideal analyte because of the typically high sensitivity of dyes to oxygen quenching. In that work, the authors showed opportunities and challenges of that type of sensor. The principle of operation relies on the emission of light by the OLED via electroluminescence (see Section 2). The light is then absorbed by the sensing element, thus re-emitted at red-shifted wavelengths and eventually detected by the photodiode. The luminescent quantum yield of the sensing element is reduced by the presence of oxygen. It is evident that an effective overlap of the spectral characteristics of the components is fundamental to ensure high sensitivity and as low as possible LOD of the sensor. To solve this issues, Liu et al. integrated (i) OPDs with a more selective spectral response, (ii) a microcavity OLED with increased emission intensity due to a more efficient light outcoupling and (iii) a more effective sensing element [156] (Figure 9).

As shown in Figure 9a, two back-to-back glass slides respectively incorporating (i) a microcavity OLED and (ii) two back-detecting OPDs were attached. The sensing element was placed on the back of the OLED substrate and was improved in terms of efficiency of oxygen detection by optimizing the ratio between polyethylene glycol (PEG) and polystyrene (PS), in which the luminescent PtOEP is embedded. The spectral characteristic of the OLED (Figure 9b) is suitably narrow to favor a good level of sensitivity of the sensor. Moreover, the emission of the sensing element matches the absorptive spectral characteristics of the photodetector, that is based on a mixture of CuPc and C_70_. For the sake of comparison, the EQE, i.e., the photon-to-charge conversion efficiency, of a spectrally mismatched OPD, based on poly(3-hexylthiophene) P3HT and *6,6*-phenyl-C_61_-butyric acid methyl ester (PCBM), is reported in Figure 9b. Remarkably, despite a high EQE of the OPD is generally desired, as in the case of the P3HT:PCBM-based OPD, the major contribution to the overall level of sensitivity arises from the spectral overlap with the OLED electroluminescence spectrum. Hence, CuPc:C_70_ is evidently preferred to P3HT:PCBM, as absorptive layer.

**Figure 10:**
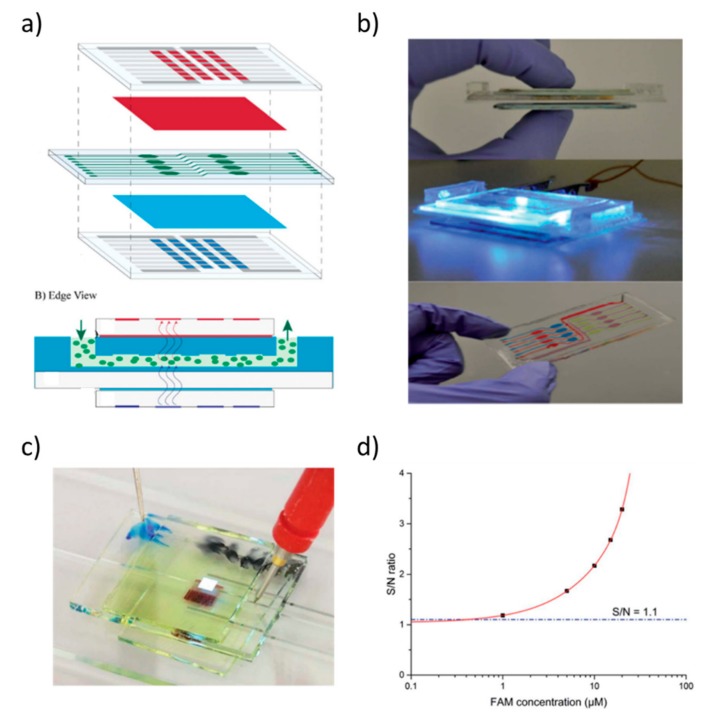
Schematic representation (**a**) and picture (**b**) of the algal fluorescence sensor based on a microfluidic chip comprising: (i) OLED, (ii) OPD, (iii) emission filter and (iv) excitation filter, adapted from Ref. [158] with permission from The Royal Society of Chemistry, © 2020. On-chip fluorescence sensor based on OLED and OPD (**c**) and the corresponding response to increasing concentration of fluorescein amidite (**d**), reproduced with permission from Ref [159], © 2020 The Royal Society of Chemistry.

By following this approach, a multitude of integrated sensors based on fluorescence detection have been realized over the years [157]. Lefevre and coworkers demonstrated a portable sensor complete with microfluidic system that is based on OLEDs and OPDs in a sandwiched structure [158] (Figure 10a,b). The sensor is designed to detect algal fluorescence and additional excitation and emission filters are introduced in the detection scheme.

Shu et al. reported on the first fluorescence light detector based on fully solution-processed organic electrochemical cells (OLECs) and an OPD for the detection of fluorescein amidite [159] (Figure 10c,d). Merfort and coworkers showed the monolithic integration of OLEDs and a-Si:H multispectral photodiodes for the detection of multiple dyes [160].

Miniaturized and disposable lab-on-a-chip devices have been realized within the European project “PHOTO-FET—Integrated photonic field-effect technology for bio-sensing functional components, Grant Agreement no. 248052” by using multifunctional field-effect transistors, in place of diodes, both as light source and detector. The aim of the project was the development of a miniaturized photonic device endowed with a microfluidic cartridge for quantitative bio-sensing for monitoring cardiovascular markers such as myoglobin and troponin-I.

Despite photoluminescent sensors comprising a transmission-type detection scheme having been successfully realized [161], the face-to-face architecture in which light source and light detector are vertically stacked on each other typically represents a limitation for the sensitivity of the sensor. Indeed, the direct illumination of photodetector by the light source represents the majority of the overall signal. Hence, excitation and emission filters are typically introduced in face-to-face photoluminescent architectures in order to improve the signal-to-noise ratio. Nevertheless, filters render the equipment relatively bulky as well as increasing the level of complexity of the system.

Over the years, the progressive engineering of the sensor architecture succeeded in avoiding the need of additional optical components. Indeed, independently from the modality of signal transduction, the relative positioning of the three sensor components (i.e., light source, light detector and sensitive element) was suitably modified to simplify the fabrication protocol and to improve the sensing performance. For instance, the monolithic, planar and concentric integration of an OLED and a ring-shaped OPD, allowed the production of a filter-less miniaturized oxygen sensor [162].

A fascinating approach to detection was developed by Ramuz et al. by evanescently coupling the light emitted by a polymeric LED (PLED) to a single-mode waveguide towards an array of polymer PDs (PPDs) [163]. The guided light interacts with the analyte, that is the mouse immunoglobulin G. While the waveguide’s surface is functionalized with the mouse immunoglobulin G, the anti-mouse immunoglobulin G is injected via microfluidic system in conjunction with Cy5, that acts as luminescence quencher. The use of different detection schemes for the integration of optoelectronic components opened towards new sensing concepts such as the combination of the absorption/transmission signal transduction with a reflection-mode detection scheme. The working principle of the sensor is summarized well in the proximity sensor that Bürgi and coworkers developed in 2005 [164]. They monolithically integrated PLEDs and PPDs, in which the light reflected by an external object is then driven back to the PPD, where is absorbed and converted into a photocurrent. The proximity of the external object tunes the intensity of light reaching the PPD, and hence the generated photocurrent.

**Figure 11:**
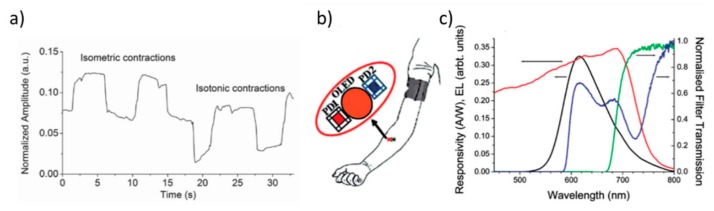
Response to isometric and isotonic muscle contractions reported by the wearable muscle contraction sensor based on the integration of one OLED and four OPDs (**a**). Representation of the bendable blood oxygenation sensor (**b**) and the corresponding electroluminescence (EL) spectra (left axis) of OC_1_C_10_-PPV-based OLED (black line), responsivity of PTB7/PC_70_BM photodiode (red line) together with their filter transmissions (right axis), i.e., 610 nm (blue line) and 700 nm (green line) (**c**). Adapted with permission from [67], © 2020 WILEY-VCH Verlag GmbH & Co. KGaA, Weinheim.

The use of that type of signal transduction and chip architecture found attractive applications in medicine. Bansal et al. recently combined OLEDs and OPDs to produce a wearable sensor for continuous health monitoring [67]. In particular, two different applications have been demonstrated. One is a muscle contraction sensor able to detect and distinguish between isotonic and isometric types of muscle contraction (Figure 11a). The second sensor consists of a bendable organic optoelectronic chip, that measures the oxygenation of human tissues. In the first case, an OLED, including Superyellow as emissive layer, has been integrated with 4 OPDs based on Poly [[4,8-bis[(2-ethylhexyl)oxy]benzo[1,2-b:4,5-b′]dithiophene-2,6-diyl][3-fluoro-2-[(2-ethylhexyl)carbonyl]thieno[3,4-b]thiophenediyl]] (PTB7): *6,6*-Phenyl-C_71_-butyric acid methyl ester (PC_70_BM) as absorbing compounds. In the latter application, the chip aims at monitoring the blood oxygenation by using hemoglobin and cytochrome aa_3_ oxidase (Figure 11c). Compared to the first application, the emissive layer of the OLED has been replaced by OC_1_C_10_-PPV polymer (Figure 11b).

**Figure 12:**
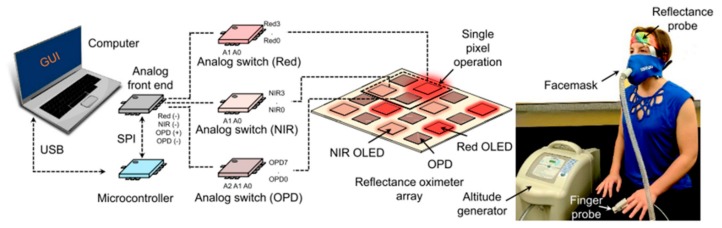
Design of the reflectance oximeter system, where each pixel comprising one red OLED, one near-infrared (NIR) OLED and two OPDs, is connected to an instrumet for driving the devices (**left** panel). Analog switches, Arduino microcontroller and universal serial bus (USB) connection are used. Setup of an altitude simulator that modifies the oxygen content of the air is showed during the operation on a volunteer breathing via a facemask (**right** panel). © 2020 by *Proceedings of the National Academy of Sciences* (PNAS), adapted with permission from [167].

The integration of OLEDs and OPDs for medicine and biodiagnostics applications has been widely reported in the last years [165]. Several reflectance-based sensors have been fabricated for wireless monitoring of the photoplethysmogram (PPG) signal [166]. In particular, Arias’ research group, one of the leaders of this research area [161,167,168], exhaustively showed a real-life application of a printed array of OLEDs and OPDs [167]. This was demonstrated through an accurate design of (i) single components, (ii) their relative distances/dimensions, (iii) their electrical connection and (iv) the collection and the analysis of the data. As a result, a flexible and printed integration of organic devices for measuring oxygen saturation with a high-quality biosignal was achieved (Figure 12). In this context, the development of the figures-of-merit of single components and the increase of the form-factor resulted in an all-day wearable pulse oxygen sensor operating at electric power as low as 24 µW [169].

**Figure 13:**
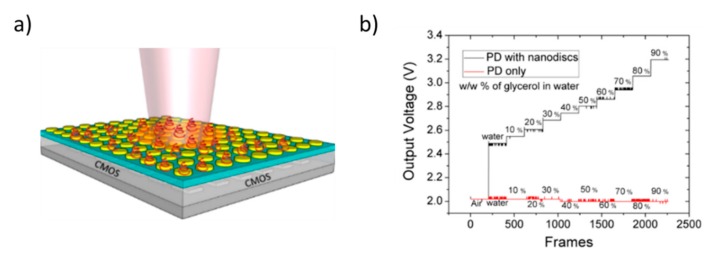
Schematic representation of a nanophotonic complementary metal oxide semiconductor (CMOS)-based biosensor (**a**) and the corresponding response at increasing glycerol concentration in water (**b**). The sensor is based on the monolithic integration of plasmonic nanostructures and a CMOS photodetector. © 2020 American Chemical Society, adapted with permission from [172].

The interest in portable and compact sensing systems for real-setting application opened towards the realization of a plethora of platforms with different functions, designs and concepts [170,171]. Most of the sensing systems rely on the integration of two or more optoelectronic devices. In this context, a breakthrough in the integration of components for sensing has recently been achieved by Shakoor et al., through the combination of photonic and optoelectronic devices [172]. The authors demonstrated the monolithic integration of a plasmonic grating with a CMOS, acting as photodiode (Figure 13). As reported in Section 3.1, the plasmonic nanostructures are widely used for sensing small variation of the refractive index of the medium taking place at their surface since it is correlated with variation of the LSPR wavelength. The integration of the plasmonic component onto the CSOM allowed detection of different concentrations of glycerol in aqueous solution (from 0 to 90% *v*/*v*) located at the grating surface, by illuminating with an external light source at 815 nm.

Aiming at ultracompact and low-cost biosensors, the authors further developed the optoelectronic-plasmonic integration by structuring the photodiode electrical contact to obtain plasmonic nanoholes with sensitivities ≥1000 nm per refractive index unit [173].

In this context, it is worth highlighting a new detection scheme currently under development in the H2020 European project “MOLOKO, Multiplex photonic sensor for plasmonic-based online detection of contaminants in milk, Grant Agreement no. 780839”. The project aims at developing a miniaturized and label-free sensor for the detection of analytes (i.e., proteins, toxins and antibiotics) in milk through the innovative combination of photonic and optoelectronic components, that is (i) a nanoplasmonic grating, (ii) an organic light-emitting transistor (OLET), used as light source, and iii) an organic photodiode, used as light detector, that is monolithically integrated onto the OLET. The three parts (that have been described in other sections of this review) are intended to work cooperatively in a reflection-like configuration for the detection of refractive index variations as small as 10^−6^ RIU upon biological stimulus. In detail, the light emitted by the OLET impinges the plasmonic grating, which modulates the luminous signal upon biochemical inputs occurring at its back-side, and then reflected back to the OPD, where is detected. By the implementation of both a suitable biofunctionalization of the sensing surface of the nanoplasmonic grating and ad hoc engineered microfluidic cartridge, the sensor is designed to detect up to 6 analytes of interest at the same time in a multiplexing configuration.

As a matter of fact, optical sensors are definitely a new scientifically and technologically challenging tool for improving the human lifespan as well as the quality of life when wearability, lightweight, and miniaturization factors are needed in the application. Nevertheless, the massive development of miniaturized and efficient single components based on nanostructured organic and hybrid materials, which has occurred over the last few decades, provides an enormous potential for the progress of new concepts and applications of sensors.

## 5. Conclusions

In this review we focused on the recent progress, main figures-of-merit and potential of different miniaturized sensing systems based on nanostructured materials. In particular, we reported on the use of organic and hybrid nanostructured materials into optoelectronic, electrochemical and plasmonic elements for highly integrated smart sensors.

The engineering of the functional properties of nanostructured materials through their chemical and structural design, together with the design of the architecture of the single-component devices, is demonstrated to be playing a major role in the realization of effective and integrated detection schemes. Indeed, for on-site bio-diagnostics and environmental/food monitoring purposes, the application of most standard and traditional analytical techniques is in contrast with the current need for rapid, cheap, easy-to-use and portable devices.

Above all, electrochemical and optical chemical sensors are promising tools with interesting analytical features. For electrochemical sensing, the major strategy that is implemented for enhancing both sensitivity and selectivity is the surface modification by nanophase-functional materials. Nanophase materials, such as carbon allotropes, metal- and metal oxide NPs, molecularly imprinted polymers, through different physical and chemical mechanisms, or acting as binders for biological recognition elements, can strongly enhance the limit of detection and analyte affinity of electrochemical sensors.

Optical sensors generally also show a good sensitivity and selectivity. Also, with respect to electronic devices, optoelectronic devices are less affected by external electric or magnetic fields and self-interference effects. The massive development of miniaturized and efficient single optoelectronic components based on nanostructured organic and hybrid materials, which has occurred over the last few decades, has provided enormous potential for the progress of new concepts and applications of optical and optical chemical sensors. In parallel, architecture engineering succeeded in avoiding the need for additional expensive optical components, thus allowing effective miniaturization and multiplexing.

On the other hand, plasmonic and photonic components obtained by nanostructuring allow the sensitivity of optical sensors to be increased. The features of the plasmonic and photonic systems are quite complementary and a new interest emerged in combining them into hybrid multicomponent systems to take advantage of the best properties of both. As one of the main advantages, the same plasmonic/photonic detection scheme can be applied to detect a large panel of molecules, often with a label-free approach (i.e., SPR). Furthermore, a specific functionalization step with biological recognition elements enable to obtain optoplasmonic or photonic chemical sensors with a high selectivity for specific target molecules.

Finally, we highlighted that integration is a key-factor to unravel the potentiality of optical-chemical sensors in terms of disposability, reliability, miniaturization and multiplexing while providing laboratory-quality analysis. In view of developing functional sensors for real-setting applications, a smart and effective system-engineering approach is necessary for realizing fast-responding, non-invasive, broadly adaptable, potentially highly sensitive and multiplexing sensors. Such systems could be employed to overcome existing limitations in measurements which are currently used in environmental and agri-food fields and advanced biodiagnostics.
